# Complete mitochondrial genome of *Morphostenophanes yunnanus* (Zhou, 2020) (Insecta: Coleoptera: Tenebrionidae) and phylogenetic analysis

**DOI:** 10.1080/23802359.2022.2097030

**Published:** 2022-07-25

**Authors:** Yu Bai, Kang Yang, Lin Ye, Xuyuan Gao

**Affiliations:** a College of Mathematics & Information Science, Guiyang University, Guiyang, China; b College of Biology and Environmental Engineering, Guiyang University, Guiyang, China; c Guangxi Key Laboratory of Biology for Crop Diseases and Insect Pests, Institute of Plant Protection, Guangxi Academy of Agricultural Sciences, Nanning, China

**Keywords:** *Morphostenophanes yunnanus*, Tenebrionidae, mitochondrial genome, phylogenetic analysis

## Abstract

*Morphostenophanes yunnanus* (Zhou, 2020) is widely distributed from central to eastern Yunnan with distinct geographical variations in morphology. Beetles were collected in Manwan Town, and a mitochondrial genome sequence (GenBank accession number MZ298928) of this species was sequenced using the MGI-SEQ 2000 platform, assembled using NOVOPlasty v4.3.1, and characterized. The mitogenome was a circular DNA molecule of 15,690 bp with 64.710% AT content, which comprised 13 protein-coding genes, 22 tRNA genes, two rRNA genes, and one control region. The protein-coding genes showed the typical ATN (Met) and TTG (Met) start codons, except *nad1* and *cox1* (TTG as start codon), and were terminated by typical TAN stop codons. The maximum-likelihood polygenetic tree was generated using protein sequences of thirteen protein-coding regions of seventeen mitogenomes with mtREV + G + F + I with 1000 replicates under the Bayesian information criterion using MEGA 11, which showed that *M. yunnanus* was the most closely related to *M. sinicus*. This study provides essential genetic and molecular data for phylogenetic analyses of the genus *Morphostenophanes*.

*Morphostenophanes yunnanus* (Zhou, 2020) is widely distributed from central to eastern Yunnan with distinct geographical variations in morphology (Zhou 2020). It has gray-black shagreened mouth parts and dark-brown tarsi (Zhou 2020). *M. yunnanus* can be divided into seven geographical populations (Zhou 2020). The complete mitochondrial genome of *M. yunnanus* of the Manwan Town population was sequenced and characterized.

Adult *M. yunnanus* specimens were collected from Shuibatou Village (100.32963° N, 24.68407° E), Manwan Town, Yun County, Lincang City, Yunnan Province, China, on 24 September 2020, and deposited in the Insect Collection of Institute of Plant Protection, Guangxi Academy of Agricultural Sciences (http://www.gxaas.net/s.php/zwbhyjs/, Xuyuan Gao, gxy@gxaas.net) under the voucher number GIPP-20200924-001. Genomic DNA was isolated and subjected to paired-end sequencing (2 × 150 bp) of 400 bp inserts using the MGI-SEQ 2000 platform. We produced ∼11.537 Gb of raw data, of which 11.301 Gb (97.76%) were clean high-quality data using SOAPnuke version 2.1.0 (Chen et al. 2018) with default parameters. The genome was assembled *de novo* using NOVOPlasty v4.3.1 (Dierckxsens et al. 2017) with default parameters and the mitogenome of *Morphostenophanes sinicus* (MW853764.1) (Bai, Gao, et al. 2021) as a seed sequence (no extending the seed directly). Three pairs of primers were designed corresponding to the *cox1*, *nad5*, and control regions (CR, also an AT-rich region) to verify the accuracy of the genome assembly. PCR products were sequenced from other *M. yunnanus* specimens using Sanger sequencing using an ABI 3730 automatic sequencer and assembled manually (Supplementary 1, 2), and only a few bases were found to be different (Supplementary 1).

The circular mitogenome (nucleotide composition: 37.935% A, 26.775% T, 23.391% C, and 11.899% G; and 64.710% AT content) of *M. yunnanus* (MZ298928.1) was 15,690 bp in length. Using Perna and Kocher’s formula (Perna and Kocher 1995), The AT and GC skews of the major strand of the mitogenome were estimated to be 0.172 and −0.326, respectively. MITOS (http://mitos.bioinf.uni-leipzig.de/) (Bernt et al. 2013) was used for the sequence annotation, revealing 13 protein-coding genes (PCGs), 1 CR, 22 tRNA genes, and two rRNA genes. Using the genomes of *Promethis valgipes valgipes* (MW201671.1) (Bai, Chen, et al. 2021), *Tenebrio obscurus* (MG739327.1) (Bai et al. 2018), *M. sinicus* (MW853764.1) (Bai, Gao, et al. 2021), *Blaps rhynchoptera* (MN267802.1) (Yang et al. 2019), and *Zophobas atratus* (MK140669.1) (Bai et al. 2019) as a reference, the start and stop codons of PCGs were corrected manually. All 13 PCGs had traditional ATN (Met) start codons, except for *nad1* and *cox1* (TTG): *atp8* starts with an ATC start codon; two PCGs (*nad3* and *nad6*) start with an ATA start codon; five PCGs (*atp6*, *cox3*, *nad4*, *nad4l*, and *cob*) start with an ATG start codon; three PCGs (*nad2*, *cox2*, and *nad5*) start with an ATT start codon. All 13 PCGs using traditional TAN as stop codons: six PCGs (*nad2*, *cox1*, *nad4*, *atp6*, *nad6*, and *cob*) ended with a TAA stop codon; five PCGs (*cox2*, *atp8*, *nad3*, *nad4l*, and *nad1*) ended with a TAG stop codon; two PCGs (*cox3* and *nad5*) had an incomplete stop codon (T), consisting of a codon that was completed by the addition of A nucleotides at the 3′ end of the encoded mRNA. The 22 tRNA ranged from 60 (tRNA-Cys and tRNA-Gly) to 72 bp (tRNA-His). The lrRNA and srRNA were 1195 and 756 bp in length, respectively. The CR of the mitogenome was 1121 bp with a 79.572% AT content, which was located between the srRNA and tRNA-Ile genes.

For phylogenetic analyses, *M. yunnanus* mitochondrial PCGs and 16 other insect species were used to construct a maximum-likelihood (ML) phylogenetic tree. Protein sequences on thirteen protein-coding regions of their mitogenomes were aligned using MEGA 11 software (Tamura et al. 2021) with MUSCLE program (Edgar 2004) with default specifies. The ML Model with the lowest Bayesian Information Criterion (BIC) scores is considered to be the best. According to BIC (=93859.54), General Reversible Mitochondrial (mtREV24) + a discrete Gamma distribution (G) + amino acid frequencies (F) + evolutionarily invariable (I) with 1000 replicates was chosen to construct a phylogenetic tree using MEGA 11 (Tamura et al. 2021) (Figure 1). Sixteen mitogenomes of species belonging to the family Tenebrionidae and the mitogenome of *Lepisma saccharina* (Bai et al. 2020) were selected as the outgroup. The resulting polygenetic tree revealed that *M. yunnanus* was the most closely related to *M. sinicus*. Overall, our study provides insights into the mitogenome of *M. yunnanus* and essential genetic and molecular data for phylogenetic analyses of the species belonging to the genus *Morphostenophanes*.

**Figure 1.**
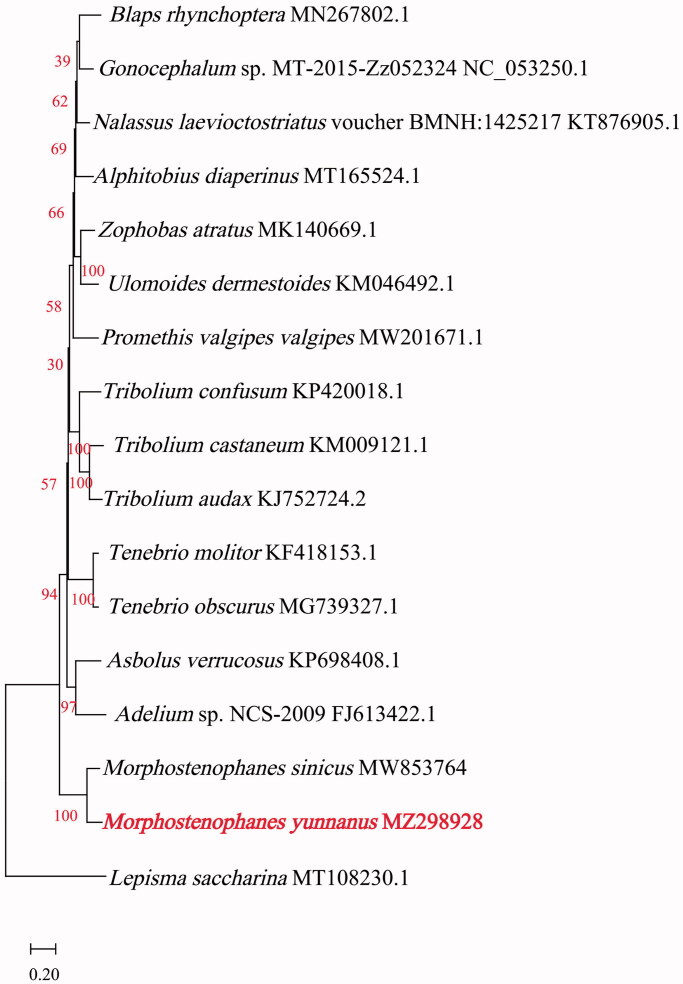
Maximum-likelihood phylogenetic tree of *Morphostenophanes yunnanus* and those of 16 other insect species based on protein sequences of thirteen protein-coding regions of their mitogenomes.

Phylogenetic relationships were inferred using maximum likelihood and mtREV + G (parameter = 0.4735) + F + I (17.50% sites). The highest log-likelihood of the tree is −48283.84. The percentage of trees is shown below the branches in red. The complete mitogenome of *M. yunnanus* (GenBank accession number MZ298928) determined in this study is indicated in red.

## Supplementary Material

Supplemental Material

## Data Availability

The genome sequence data that support the findings of this study are openly available in GenBank of NCBI at https://www.ncbi.nlm.nih.gov under the accession no. MZ298928. The associated BioProject, SRA, and Bio-Sample numbers are PRJNA729642, SRR14523735, and SAMN19159615, respectively.
