# Supplementary material for: Complete mitochondrial genome of Morphostenophanes yunnanus (Zhou, 2020) (Insecta: Coleoptera: Tenebrionidae) and phylogenetic analysis
Source: Mitochondrial DNA B Resour. 2022 Jul 25;7(7):1352–4. doi: 10.1080/23802359.2022.2097030 (PMC9318264; doi:10.1080/23802359.2022.2097030)
Supplement: Supplemental Material [file TMDN_A_2097030_SM2101.docx]

**Supplementary 1 Sequence validation for mitogenome of *Morphostenophanes yunnanus* (MZ298928) using Sanger method**

Design 3 pairs of specific primers based on the mitogenome sequence of *Morphostenophanes yunnanus* for direct PCR amplification. The PCR reaction was carried out with LA Taq polymerase for 35 cycles at 94°C for 30 s, and annealed at 50°C for 30 s, followed by extension at 72°C for 1 min per 1 kb. The final MgCl2 concentration in the PCR reaction was 2.0 mmol/L. PCR products were cloned into pMD18-T vector (Takara, JAP) and then sequenced, or sequenced directly by the dideoxynucleotide procedure, using an ABI 3730 automatic sequencer. Sequences were assembled by software of DNAstar and adjusted manually to generate the complete sequence of mitochondrial DNA.

**The bases in red color in the sequence are different from those in NGS sequencing**

**1. *cox1***

**Primer sequences:**

DZ COX1F: CCACGCAAGAACCAAAGCCT

DZ COX1R: GACGCTCTGTCTTGAAGAAGC

PCR products from *cox1* were cloned into pMD18-T vector (Takara, JAP) and then sequenced. We obtained ZL-COX1-7_M13R-48_TSS20210926-027-09466_D08 and ZL-COX1-7_M13F-47_TSS20210926-027-09466_G08.

PCR products from *cox1* were sequenced directly by the dideoxynucleotide procedure. We obtained 0001_31422041600058_(ZL-COX1)_[DZCOX1F] and 0002_31422041600059_(ZL-COX1)_[DZCOX1R]

>ZL-COX1-7_M13R-48_TSS20210926-027-09466_D08, ZL-COX1-7_M13F-47_TSS20210926-027-09466_G08

CCACGCAAGAACCAAAGCCTAAAGATTTCATACCTTTAAACTTGCAATTTAATATCATACTTGACTATTAGGCCAACCAATAACAGTGGAAATTTACTCATGAATAGATTTACAGTCTATTGCCTAATTCAGCCACACTACCGAATAAGTGACTATTCTCAACCAACCACAAAGACATTGGAACACTTTACTTTATCTTTGGAGCCTGATCAGGAATAGTGGGAACATCGCTAAGAATGATAATCCGGGCAGAACTAGGCAACCCTGGTTCTCTCATCGGAGACGACCAAACCTACAACGTAGTCGTAACCGCACACGCTTTCGTAATAATTTTTTTCATAGTAATGCCTATTATAATCGGCGGATTCGGAAACTGGCTAGTACCTTTAATATTAGGCGCCCCTGATATAGCTTTCCCCCGTATAAATAACATAAGCTTTTGACTACTTCCACCATCTCTCTCACTTTTACTAATAAGAAGAGTTGTAGAAAACGGAGCAGGAACCGGACGAACAGTGTACCCCCCACTATCATCTAGTATTGCTCACAGTGGCTCCTCAGTCGACCTCGCAATCTTTAGACTTCACCTAGCTGGAATCTCATCAATTCTAGGTGCCGTAAATTTCATTACCACAGTAATCAATATACGACCCCAGGGAATATCATTAGACCGAATACCTCTCTTCGTATGGGCAGTAGTAATCACTGCAGTACTATTACTCCTGTCACTCCCTGTTCTTGCAGGCGCAATCACCATGCTACTAACTGACCGCAATATTAATACATCCTTCTTTGACCCCGCAGGAGGAGGAGACCCAATTCTTTACCAGCATCTATTCTGATTTTTCGGACATCCTGAAGTTACATTCTCATTCTACCAGGGTTTGGCATGATCTCTCACATTATCAGCCAAGAAAGAGGGAAAAAGGAAGCCTTCGGAACCCTGGGAATGATTACGCAATAATAGCAATCGGCTTGCTTGGATTCGTAGTATGAGCACACCACATATTCACTGTAGGAATAGACGTTGACACCCGAGCATACTTCACATCCGCCACCATAATCATTGCTGTACCTACAGGAATCAAAATTTTCAGCTGATTAGCTACCCTTCACGGAACCCAGTTAACATACAGCCCCTCACTTCTGTGAGCACTTGGATTTGTATTCTTATTCACAGTAGGTGGTCTCACTGGTGTCGTACTAGCAAACTCTTCTATTGACATTATACTACACGATACCTATTATGTAGTAGCCCACTTCCACTACGTTCTATCCATAGGCGCAGTATTTGCCATCATAGGAGGCCTAATTCACTGATACCCTCTATTTACAGGCCTTTCATTAAGGCCTAAATTATGCAAGATTCAATTTTTAACTATATTCATTGGAGTAAATTTAACTTTCTTTCCACAGCACTTCCTAGGATTAAGCGGGATACCCCGCCGATATTCAGACTACCCCGATGCATACACCTTATGAAACATCGTTTCCTCTGTAGGATCCATCATTTCCCTTATCGGAGTACTACTTCTAGTATTTATCGTATGAGAAAGATTCTCAGCCGCACGAAAAACACTTATTCCCCTTAACATGACCGCATCAATCGAATGACTACAATCAACACCGCCCGCTGAACATAGCTATTCTGAGCTTCCTATACTAACATCTAGTTTCTAATGTGGCAGAATAGTGCGGTGGACTTAAGCCCCAATTATAAAGATTTCTCTTTCTTTAGAAATTGCAACCTGAAAACTCACGCTTCTTCAAGACAGAGCGTC

>0001_31422041600058_(ZL-COX1)_[DZCOX1F], 0002_31422041600059_(ZL-COX1)_[DZCOX1R]

TAGGACTCTACTTGCATTTAATATCATACTTGACTATTAGGCCAACCAATAACAGTGGAAATTTACTCATGAATAGATTTACAGTCTATTGCCTAATTCAGCCACACTACTGAATAAGTGGCTATTCTCAACCAACCACAAAGACATTGGAACACTTTACTTCATCTTTGGAGCCTGATCAGGAATAGTGGGAACATCACTAAGAATGATAATCCGGGCAGAACTAGGCAACCCTGGTTCTCTTATCGGAGACGACCAAACCTACAACGTAGTCGTAACCGCACACGCTTTCGTAATAATTTTTTTCATAGTAATGCCTATTATAATCGGCGGATTCGGAAACTGACTAGTACCTTTAATATTAGGCGCCCCTGATATAGCTTTCCCCCGTATAAATAACATAAGCTTTTGATTACTTCCACCATCTCTCTCACTTTTACTAATAAGAAGAGTTGTAGAAAACGGAGCAGGAACCGGATGAACAGTGTACCCCCCACTATCATCTAATATTGCTCACAGTGGCTCCTCAGTCGACCTCGCAATCTTTAGACTTCACCTAGCTGGAATCTCATCAATTCTAGGTGCTGTAAATTTCATTACCACAGTAATCAATATACGACCCCAGGGAATATCATTAGACCGAATACCTCTCTTCGTATGGGCGGTAGTAATCACTGCAGTACTATTACTCCTATCACTCCCTGTTCTTGCAGGCGCAATCACCATGCTACTAACTGACCGCAATATCAATACATCCTTCTTTGACCCCGCAGGAGGGGGAGACCCAATTCTTTACCAGCACCTATTCTGATTTTTCGGACATCCTGAAGTTTACATTCTCATTCTACCAGGGTTTGGTATAATCTCTCACATTATCAGCCAAGAAAGAGGAAAAAAGGAAGCCTTCGGAACCCTAGGAATGATTTACGCAATAATAGCAATCGGTTTGCTTGGATTCGTAGTATGAGCACACCACATATTCACTGTAGGAATAGACGTTGACACCCGAGCATACTTCACATCCGCCACTATAATCATTGCTGTCCCTACAGGAATCAAAATTTTCAGCTGATTAGCTACCCTTCACGGAACCCAGTTAACATACAGCCCCTCACTTCTGTGAGCACTTGGATTTGTATTCTTATTCACAGTAGGAGGTCTCACTGGTGTCGTACTAGCAAACTCTTCTATTGACATTATATTACACGATACCTATTATGTAGTAGCCCACTTCCACTACGTTCTATCCATAGGCGCAGTATTTGCCATCATAGGAGGCCTAATTCACTGATACCCTCTATTTACAGGCCTTTCATTAAGGCCTAAATTATGCAAGATTCAATTTTTAACTATATTCATTGGAGTAAATTTAACTTTCTTTCCACAGCACTTCCTAGGATTAAGCGGGATACCCCGCCGATACTCAGACTACCCCGATGCATACACCCTATGAAACATCGTTTCCTCTGTAGGATCAATCATTTCCCTTATCGGAGTACTACTTCTAGTATTTATCGTATGAGAAAGATTCTCAGCCGCACGAAAAACACTCATTCCCCTTAACATGACCGCATCAATCGAATGACTACAATCAACACCACCCGCTGAACATAGCTATTCTGAACTTCCTATACTAACATCTAATTTCTAATGTGGCAGAATAGTGCGGTGGACTTAAGCCCCAATTATAAAGATTCTCTTCTTAGAAATGCATCCGAC

**2. *nad5***

**Primer sequences:**

DZ ND5F: CAGTGTAGATTTAGCTAAGG

DZ ND5R: GTAGGTTGCGGGAGTATCTG

**>ZL-NDS-20_M13R-48_TSS20210927-027-09759_C08, ZL-NDS-20_DZW125R1_TSS20210927-027-09759_A08, ZL-NDS-20_M13F-47_TSS20210927-027-09759_B08**

CAGTGTAGATTTAGCTAAGGCTTATAAATACCTGAAAATATTAATCTCCTTGATATCTTCAATATCACGCTCTACATAAGCTATCCAAGCAAAACATAAAAACAAGAAAAAACCACACAACTATTAACACAAAGAAAACCTTAATACTATTAAACATTACCAATTGCACCAATGTCGACTTGCCCCTCAAAATCATATAAATCTTTTGAGCCCCAAAAAACTCTGCCCAACCATGGTCAAGGGATTTGAACATAGCTGAACCCCCCACTAAAGGATAGTACCTAACTCCTAACGTTGAAATAACAGGTATATTCCACATCCCCCTAAAAAACCACGAAAGCTTATACCTTTTTAAAGAAAGGTTAACTCGGCTAAAATCAAACTTAGCTAACTCATAGCCCAAGTACATTCCCAAAAAAATAAACAATAAAGCAGTCACCTTCATAAAAGTAGGGAGAATGATAACATACGGAAAAGGAAAAATAAGCCAACATAACAGCCTACCTATAAACACTACAAAAAAAATTAACCCTGTTATGCCACGTAACATTATCGAACCAGATTCCCTCACCGACCTTATAGCCCCATAATTACCATTACCCACAAATCCATAATAAACCAACCGAAACCTATAACAGACGGTTAAACCTAAAGACAAATAAAAAATCATATAAATATAGCCACCAACATACCCCATGCTCATAACCTCAGCAACCAAGTCCTTAGAGTAAAACCCTCTAAGAAACGGTAGGCCACATAAAGCTAAATTACAAATATTAAAATACCTAGAAACAATGGGCATACCCGTACCTAACCCCCCCATACAACGAATGTCTTGGACATTACCCATTGAATGAATAATACTACCAGCACACATAAACAACAAAGCCTTAAACAAAGCATGCACCAGCAAGTGAAAAAACGCCAGCTTATAGCCGCCTAAGGACAAAACTATAATCATAAGACCAAGCTGACTAAGAGTAGACAAAGCAATAATTTTTTTCAAGTCAAATTCAAAGCTAGCCCCTAAACCAGATATAAACATAGTCATCCTAGAAATAAACAATAAAAAATACACAGCATTTTCACCCAAAGCATAATTAAACCGAATAAGCAAGTAAACCCCTGCAGTCACTAATGTAGAAGAATGAACCAAGGCCGAAACCGGCGTAGGAGCCGCCATAGCAGCAGGCAGCCATGAAGAAAATGGGATCTGAGCACTCTTAGTCATTCCTGCTAAAATTACAAGTCAAGTAACAAAAACTATGCTTTTGTCACCCTTTAACTCCTCCAAGTAAAATACATAGTTCCAACTACCAAAATTTAGTATCCAAGCAATCGCCATCAAAAGCGCCACATCACCCAAACGATTGCTTAAAGCTGTTAATATCCCAGCCCTAAAAGACTTAACATTTTGATAATAAATAACCAAACAATAAGAAACCAGGCCCAGCCCATCCCAGCCCAATAAAATACTAATTAAATTAGGGCTAACAATAAGCAAAACCATAGAAAGAACAAACATAGCAACCAAAATAATAAAACGATTAATGTACAAATCTCCTGCCATATACTCCTGCCTATAATACACTACTATGCCTGAAATAATCAAAACAAACCTAATAAACACAAGCGATATCCAGTCAAAATAAAAAGTCATAACAATTCCGCCCGAATTCATTATAACAAGATTATACTCAAAAAAAACGCTATAATCTAAAGCTAAAAAATAAACCCCCAAAATAAACAATGCCACACTAATACTACCAAATAAACCAAAATAAATAACACAAATATTAGGCAATATTTAAAATACTATAAATATATCCCTGACCCCACAAATCAGTGTTTTACTTAAACTACTTAAATAAAATCATAAAACAACCCAGCCTCCTGTCAAAACCACAACATTTAAGGGAATTCAATGCAACCCTAAAACCAGATACTCCCGCAACCTAC

**3. Control Region**

**Primer sequences:**

DZ DLF: CCAAGTCTAGAATCCCCTAATAC

DZ DLR: GTTTTAGTATAGGATGTACAAGG

**>ZL-DL-44_M13F-47_TSS20210924-027-08586-01_G06, ZL-DL-44_DZDLR1_TSS20210924-027-08586-02_D04, ZL-DL-44_M13R-48_TSS20210924-027-08586_G07**

CCAAGTCTAGAATCCCCTAATACTTAAAACTACATACTACAAAATCCCCTATAAAATCAATATGCCTTATATATAAACACAAACATTTAGCTAGCTTTTAAGCCAAAATAAAACTTTGGCACAACCACAACAGACACTAACCCCATATCAACTATCTCCCGCAGAATAGCGCTCTCCCCCAAGAGCAGTTTTTTAGCTATCCGTAATTTAGAGTACCAAGAAAACCCCCTCCGTTAGCACCCCCCAGTGCCGACTAATTTTTTTTTTTTTACTGTAAAATTACTATTCACTAACCCAACCCCGGCTCGCGCCGCCGCGTATAGTATTATTTATTTGATTATAAAGAGTTTAATTACTAAAACATTAAACAGTAATATTACTATATTAGTCTATTATCATAAAATTACTATATACTATAATAGCAATGAAAGTTAAATATACTCTCTAACCTTATTCTATAAGACTATTTATTATACGATAGAAATAAGGGTTTTTTTTTTTTTTTCATTAAATATTTATATATTATTATATATATAGATATATAAGGATATATTAATATATATATATTTATATATTATAATTTATATAATAATAAATTAAATCATTATATATATAACAATATTATATATATATATATTAAATATTTATATATATATATTATATATGATTATAGATATATAAGAATTTAATATTATATAAAATATTTATATAATATTATTATATAATTCTTATATATCTTCTTTTTCTTTTTTTTTTTAGAGTCTTTACTATACTATAAGCTGTATTGATAAATTGGTATTATATACATATTAAACCAGATTATATATATATATAAAATAATATATAAATTATAGTAATATTATATAAACATTATATATCTATCGCTTATTTATATATAATGTATATATATATATAAGAATTTAATATATAATATATAATATATATTCTTGGATTCAACAAGTTTTAATATAGAGCAGAAAATAAAATTGACTTTACATTTTTATTTTTCCCAATAAATTAAACACCCTAGTTAAATAATTCTTGTACAAAAATGCCCTCATAAGACGATTTTCGAAAAAACAGTACTAAAAAAACTTAGTCTAATCAAATCCTCGAGAAAAAAAAAAGTGTTATTTTTTTTATTCATTTTTTTTCAAAATTCCTAAATTAAAATTAACTACCAATCTACCTTCTTAAATACATATTAGGTATAGTAAAAACTAAATTTTATAATAAAGTTTTAATGAAATGCCTGAAAAAGGATTACTTTGATAGAGTAAATTATACACTCCCGTGTTTTCATTACATTTGACAGACCCAATCCGCCCCCTAGGGTACCAAAAACCCTTGTACATCCTATACTAAAAC
